# Temporalis muscle flap in craniofacial reconstruction: anatomy, techniques, outcomes, and innovations

**DOI:** 10.3389/fsurg.2025.1678935

**Published:** 2025-10-09

**Authors:** Ingrid C. Landfald, Teresa Vazquez, Adrian Okoń, Łukasz Olewnik

**Affiliations:** 1 Department of Anatomical Dissection and Plastination, Mazovian Academy in Płock, Płock, Poland; 2 VARIANTIS Research Laboratory, Department of Clinical Anatomy, Mazovian Academy in Płock, Płock, Poland; 3 Donor Body Center, Complutense University of Madrid, Madrid, Spain

**Keywords:** temporalis muscle flap, craniofacial reconstruction, vascular anatomy, facial reanimation, temporal hollowing, surgical anatomy, virtual surgical planning, dynamic muscle transfer

## Abstract

**Background:**

The temporalis muscle flap (TMF) remains an essential reconstructive option in contemporary craniofacial reconstructive surgery (CRS) owing to its reliable vascularity, anatomical proximity to common defect areas, and substantial soft tissue volume. Despite extensive historical use, evolving surgical approaches and novel adjunctive technologies necessitate an updated comprehensive review to guide current clinical practice.

**Objective:**

This review critically examines the TMF regarding its anatomical considerations, surgical innovations, clinical outcomes, and functional restoration capacities. Additional objectives include a detailed assessment of clinical complications, identification of existing gaps in knowledge, and evidence-based comparisons with alternative reconstructive techniques.

**Methods:**

An extensive literature review was conducted utilizing current high-quality publications, including systematic reviews, clinical series, cadaveric anatomical studies, and reports detailing innovative techniques from major surgical journals. Specific emphasis was placed on the latest minimally invasive, endoscopic, and robotic-assisted approaches, alongside novel tissue engineering methodologies and virtual surgical planning (VSP). Clinical outcomes, complication rates, patient satisfaction levels, and comparative analyses with alternative reconstructive flaps, including free tissue transfers and other regional flaps, were rigorously assessed.

**Conclusion:**

TMF remains a versatile, robust, and highly reliable reconstructive option within modern craniofacial surgery. Anatomical knowledge, meticulous surgical technique, and incorporation of emerging adjunctive technologies significantly enhance outcomes while minimizing morbidity. Continued research into minimally invasive techniques, regenerative medicine, functional restoration through advanced nerve transfers, and secondary refinement procedures is essential to further improve clinical efficacy, patient satisfaction, and overall quality of life.

## 1 Introduction

The temporalis muscle flap (TMF) represents a well-established and anatomically robust reconstructive option within the domain of craniofacial surgery, with historical descriptions dating back to the late nineteenth and early twentieth centuries (1–3). Its enduring clinical relevance derives from a unique combination of anatomical proximity to midfacial and skull base regions, substantial muscular bulk, and highly reliable vascularity, rendering it particularly effective in managing defects following trauma, ablative oncological procedures, and congenital anomalies, as well as in dynamic and static facial reanimation (4–6).

Anatomically, the TMF receives a dual arterial supply primarily from the anterior and posterior deep temporal arteries (branches of the maxillary artery) and secondarily from the middle temporal artery, a branch of the superficial temporal artery. Given its Mathes–Nahai type III vascular pattern (see §3.1), the TMF remains highly reliable in challenging reconstructive settings, including irradiated or vessel-depleted fields (4, 5).

Technological advancements over the past two decades, including minimally invasive harvesting approaches, endoscopic and robotic-assisted dissections, and integration of virtual surgical planning, have broadened the applicability of TMF and substantially mitigated traditional complications such as donor-site morbidity, visible scarring, and iatrogenic injury to the frontal branch of the facial nerve (6, 7). Furthermore, the incorporation of regenerative adjuncts autologous fat grafting and patient-specific implants fabricated from polyetheretherketone has improved aesthetic outcomes, particularly in addressing postoperative temporal hollowing (8, 9).

Nonetheless, several limitations remain unresolved. The TMF does not provide intrinsic osseous support, limiting its use in composite maxillofacial reconstructions requiring skeletal replacement. In addition, complications such as persistent trismus or functional asymmetry continue to be reported despite meticulous technique (8, 10). These clinical challenges highlight the need for standardized anatomical protocols, improved outcome reporting, and comparative studies with microvascular and regional alternatives.

This review aims to deliver a comprehensive synthesis of the current anatomical, surgical, and clinical evidence on TMF, critically analyzing its historical evolution, modern applications, and future directions. Emphasis is placed on its anatomical basis, reconstructive versatility, and the integration of advanced imaging and minimally invasive strategies to optimize both functional and aesthetic outcomes.

## 2 Historical development of the temporalis muscle flap

### 2.1 Early descriptions

The earliest known application of the TMF in surgical practice was reported by Lentz in 1895 (11), in the context of temporomandibular joint ankylosis. This was soon followed by Golovine's adaptation for orbital reconstructions in 1908, marking the beginning of its broader reconstructive potential. A pivotal contribution came from Sir Harold Gillies, who, during and after World War I, systematically employed the TMF for major cheek and midfacial defects, thus establishing foundational principles for soft tissue reconstruction that remain relevant in modern craniofacial surgery (2, 3) [Lewis 1910; Gillies 1920 (12), cited in Clauser et al. (13)].

### 2.2 Mid-20th century refinements

In the mid-twentieth century, the reconstructive utility of the TMF was further enhanced by the introduction of static sling techniques by Gillies (12) and later McLaughlin (14). These methods facilitated facial symmetry restoration in cases of long-standing facial paralysis, broadening the clinical indications of the flap and laying the groundwork for subsequent dynamic applications (13).

### 2.3 Late 20th century innovations

A significant paradigm shift occurred in 1997 with the introduction of lengthening temporalis myoplasty (LTM) by Labbé (15), who utilized fascia lata grafts to enable dynamic smile restoration in a single-stage procedure. This technique proved to be a less invasive and more accessible alternative to free gracilis muscle transfer, demonstrating favorable functional and aesthetic outcomes and thus reaffirming the TMF's value in facial reanimation (15, 16).

### 2.4 Expanded applications and recent advances

Since the emergence of LTM, the indications for TMF have expanded considerably, encompassing orbital, maxillary, skull base, and oral cavity reconstruction. These broader applications have been facilitated by advancements such as minimally invasive harvesting techniques, endoscopic and robotic-assisted access, and preoperative planning with virtual surgical platforms. Additionally, the introduction of biomaterials including PEEK implants and autologous fat grafts has further improved reconstructive precision and patient satisfaction (6, 9).

### 2.5 Critical analysis of historical evolution

Over the past century, the TMF has evolved from a static volume replacement method to a dynamic tool for functional restoration, reflecting increased understanding of its layered anatomy, neurovascular supply, and biomechanical behavior (4, 5). Despite this progress, complications such as postoperative temporal hollowing, persistent trismus, and the inability to provide skeletal reconstruction continue to limit its broader use. Addressing these challenges will require the integration of regenerative solutions, improved imaging protocols, and comparative outcome data from prospective clinical trials (7, 8, 10).

A chronological overview of major historical milestones in the evolution of the temporalis muscle flap, from its initial application in temporomandibular joint surgery to modern tissue engineering approaches, is summarized in Table 1. This progression underscores the flap's transformation from static structural use to dynamic and precision-guided applications in facial reanimation and skull base reconstruction.

**Table 1:** Historical milestones in the development of the temporalis muscle flap.

Year	Author(s)	Key advancement
1895	Lentz	Initial use in TMJ ankylosis repair
1908	Golovine	Expanded indication to orbital defects
1920	Gillies	Static sling procedures for facial paralysis
1997	Labbé	Lengthening temporalis myoplasty (LTM) for dynamic smile reanimation
2000s	Multiple contemporary authors	Minimally invasive, endoscopic, robotic-assisted methods, tissue engineering, VSP integration

## 3 Vascular anatomy of the temporalis muscle flap

### 3.1 Classification of vascular supply

The TMF is classified as a Mathes–Nahai type III muscle flap, characterized by the presence of two independent dominant vascular pedicles (4, 5). This dual vascular arrangement significantly enhances clinical reliability, allowing for safe mobilization of the muscle even in challenging anatomical or surgical contexts. Such redundancy is of particular relevance in craniofacial reconstruction, where previous surgery, scarring, or irradiation may compromise local tissue vascularity (4, 6).

### 3.2 Primary vascular supply: deep temporal arteries

The dominant vascularization of the temporalis muscle is provided by the anterior and posterior deep temporal arteries, which originate from the internal maxillary artery a terminal branch of the external carotid system. These arteries course along the deep (periosteal) surface of the muscle and form an extensive intramuscular anastomotic network, facilitating uniform perfusion throughout the flap (4). The integrity of this deep vascular system is critical for the safe execution of muscle elevation and mobilization, especially in dynamic procedures requiring significant muscle excursion (5).

### 3.3 Secondary vascular supply: middle temporal artery

Supplementary vascularization is provided by the middle temporal artery, which arises from the superficial temporal artery and penetrates the temporoparietal fascia to reach the superficial portion of the temporalis muscle. This secondary supply enhances perfusion redundancy, particularly in flap regions distant from the deep pedicles or in cases where deeper vascular channels are surgically interrupted or compromised by prior irradiation (5, 6).

### 3.4 Clinical advantages of dual vascularity

Given the two independently perfused deep temporal pedicles (Mathes–Nahai type III; see §3.1), the temporalis muscle flap remains reliably perfused after extensive elevation, lengthening, or rotation; detailed outcomes in irradiated or vessel-depleted fields are discussed in §5.4 (4, 8, 10).

Practical implication — split TMF. The presence of independent deep temporal pedicles and the supplementary middle temporal artery enables splitting of the temporalis muscle (commonly anterior 2/3 vs. posterior 1/3) to address separate reconstructive sites in a single stage. Cadaveric and clinical data show the middle temporal artery can maintain viability of the split component, expanding indications while preserving volume (10, 17, 18). When split TMF is planned, CTA mapping of pedicles and careful intramuscular dissection along vascular territories are recommended.

### 3.5 Innervation of the temporalis muscle flap

Motor innervation of the temporalis muscle is provided by the anterior and posterior deep temporal nerves branches of the mandibular division (V3) of the trigeminal nerve. These nerves consistently enter the muscle on its deep surface, ensuring stable motor function and preservation of muscle tone after flap harvest (4). However, anatomical variation may include accessory innervation via the buccal and masseteric nerves, necessitating cautious dissection to maintain neuromuscular viability, especially in dynamic applications such as temporalis tendon transfer (19).

### 3.6 Surgical considerations: adjacent structures and clinical safety

Surgical elevation of the TMF requires a comprehensive understanding of the regional anatomy, particularly the proximity of the frontal branch of the facial nerve (FBFN), which courses within the temporoparietal fascia above the zygomatic arch (19). Inadvertent injury to the FBFN can result in functional and aesthetic deficits, including brow ptosis and facial asymmetry. Meticulous dissection in the loose areolar tissue plane between the temporoparietal and deep temporal fascia layers is essential to avoid nerve damage (10, 15).

The STA and accompanying vein, which traverse superficially within the same fascia, must also be preserved to maintain optimal flap perfusion. Furthermore, aggressive dissection at the mandibular coronoid insertion often necessary for tendon mobilization can weaken mandibular integrity and increase fracture risk, particularly in irradiated bone (6, 20).

**Figure 1:**
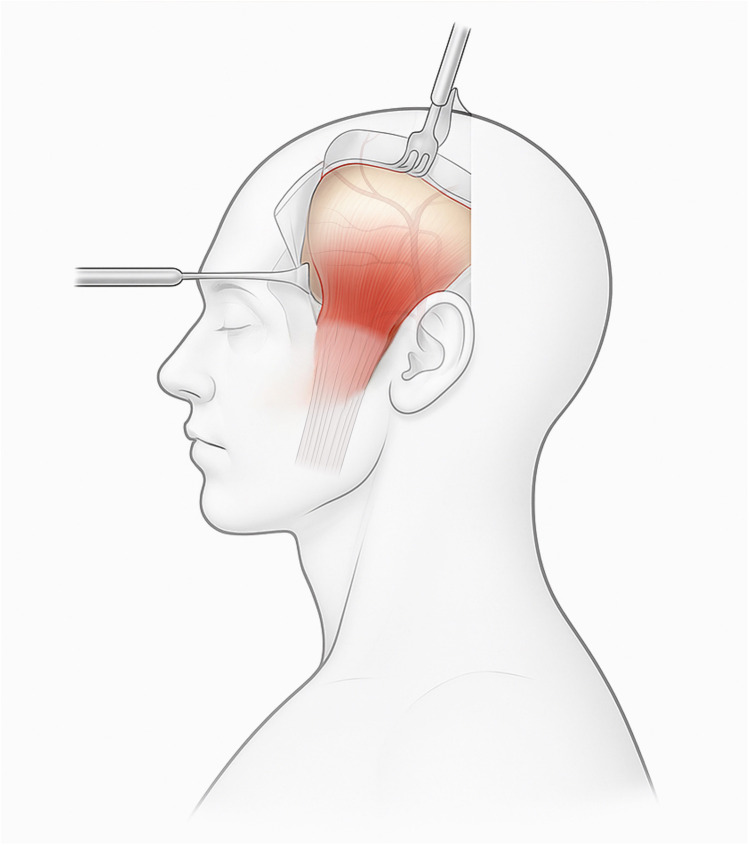
Endoscopy-assisted harvest of the temporalis muscle flap (TMF) through a limited temporal incision with transorbital endoscopic visualization of the recipient corridor. Dissection in the plane between the temporoparietal and deep temporal fasciae preserves the frontal-branch zone and superficial temporal vessels. Clinical note: maintaining the sub-TPF plane at the zygomatic arch helps prevent brow ptosis; avoid aggressive subperiosteal elevation near the coronoid to reduce postoperative trismus.

Figure 1 provides a schematic overview of TMF elevation using a hemicoronal approach, highlighting key anatomical planes and dissection vectors relevant to preserving neurovascular structures.

### 3.7 Role of preoperative imaging and intraoperative monitoring

The integration of advanced imaging modalities and real-time monitoring technologies has substantially enhanced both the precision and safety of TMF surgeries. High-resolution computed tomography angiography (CTA) allows for detailed visualization of the vascular architecture, enabling the identification of anatomical variations in the deep temporal and middle temporal arterial systems. This facilitates individualized surgical planning and minimizes the risk of intraoperative vascular compromise (6, 7).

Magnetic resonance imaging (MRI) further contributes to preoperative assessment by offering superior soft tissue resolution, particularly valuable in evaluating muscle volume, quality, and fascial integrity prior to flap harvest (5, 21, 22). The implementation of intraoperative nerve monitoring especially electromyographic mapping and direct nerve stimulation has become an essential adjunct in preserving functional neural pathways, particularly during dynamic procedures such as temporalis tendon transposition for facial reanimation (15, 19). These techniques significantly reduce the risk of iatrogenic injury to the FBFN and enhance postoperative functional outcomes.

### 3.8 Vascular and neural anatomy of the temporalis muscle flap

**Figure 2:**
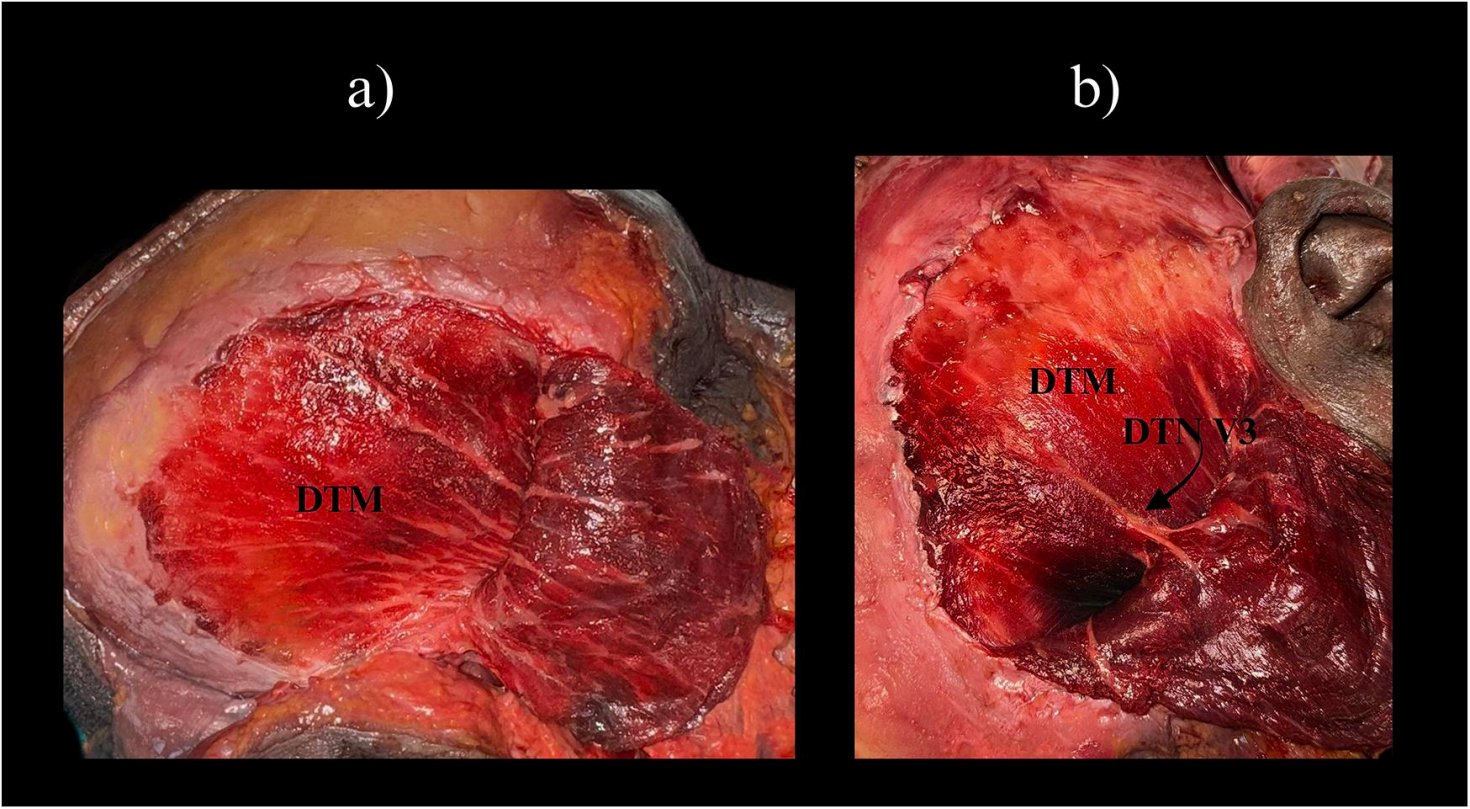
Cadaveric dissection of the temporal region demonstrating the two portions of the temporalis muscle. **(a)** Superficial temporalis muscle (STM) and the underlying deep temporalis muscle (DTM) are visible, separated by the interlaminar plane. **(b)** Similar view with exposure of the deep temporal nerve (DTN, branch of V3) at its entry zone on the deep surface of temporalis. Clinical note: preserving the DTN entry zone helps maintain muscle tone—important when planning split-TMF or dynamic temporalis transfers. STM, superficial temporalis muscle; DTM, deep temporalis muscle; DTN, deep temporal nerve; V3, mandibular division of the trigeminal nerve.

Given the anatomical complexity of the temporal region, a comprehensive understanding of the relevant vascular and neural elements is critical for safe and effective TMF elevation. The dual arterial supply via the anterior and posterior deep temporal arteries and the middle temporal artery confers high perfusion reliability across different zones of the muscle (4, 5). These vessels are typically located deep to the temporalis muscle or within the temporoparietal fascial system, necessitating meticulous layer-by-layer dissection. Figure 2 presents a cadaveric dissection of the temporalis muscle demonstrating its superficial and deep portions (STM and DTM), with clear visualization of the deep temporal nerve (DTN, V3), an essential structure for preserving dynamic function.

On the neural level, the TM receives consistent motor innervation from the deep temporal nerves (branches of CN V3), which penetrate the deep surface of the muscle in a predictable pattern (4). However, anatomical variability including accessory innervation from the buccal or masseteric nerves must be considered, especially in functional applications.

The temporoparietal fascia (TPF) plays a dual role as both a surgical landmark and a protective barrier housing the superficial temporal artery and the FBFN. Dissection within the loose areolar tissue plane separating the TPF from the deep temporal fascia (DTF) allows for safe flap elevation while preserving neurovascular structures critical to facial function and aesthetics (10).

A synthesized overview of these structures including their anatomical course and surgical significance is provided in Table 2, serving as a practical intraoperative reference for preserving functional and vascular integrity during TMF procedures.

**Table 2:** Overview of vascular and neural anatomy of the temporalis muscle flap.

Anatomical structure	Description	Clinical relevance
Anterior & posterior deep temporal arteries	Primary blood supply from branches of internal maxillary artery	Ensures robust, reliable vascular supply, flap reliability
Middle temporal artery	Supplementary superficial supply from STA	Provides additional vascular redundancy
Anterior & posterior deep temporal nerves (CN V3)	Motor innervation via mandibular nerve branches	Essential for dynamic muscle function
Frontal branch of facial nerve	Runs superficially in temporoparietal fascia	Critical nerve structure to preserve intraoperatively
Temporoparietal fascia	Protective anatomical barrier, houses vessels/nerves	Prevents nerve and vessel injury during dissection

## 4 Anatomical relationship and surgical implications of the frontal branch of the facial nerve

The anatomical trajectory of the FBFN through the TPF presents significant surgical vulnerability due to its superficial, oblique course immediately superior to the zygomatic arch (4, 10). Given its close proximity to standard dissection fields in TMF harvest, unintentional injury to the FBFN may result in serious functional deficits, including brow ptosis, impaired frontalis muscle activity, and lasting facial asymmetry (15, 19).

Meticulous dissection along the avascular loose areolar tissue plane between the superficial TPF and the deeper temporal layers is therefore critical. Adhering to precise anatomical landmarks in this region minimizes the likelihood of nerve injury and facilitates optimal functional and aesthetic outcomes (8, 10).

### 4.1 Detailed layered anatomy of the temporal region and Its surgical relevance

Successful TMF elevation demands a clear understanding of the layered anatomy of the temporal region. From superficial to deep, these layers include:

- Skin
- Subcutaneous tissue
- TPF
- Loose areolar connective tissue
- DTF
- TM
- Periosteum and pericranium of the cranial vault (4, 10).

**Figure 3:**
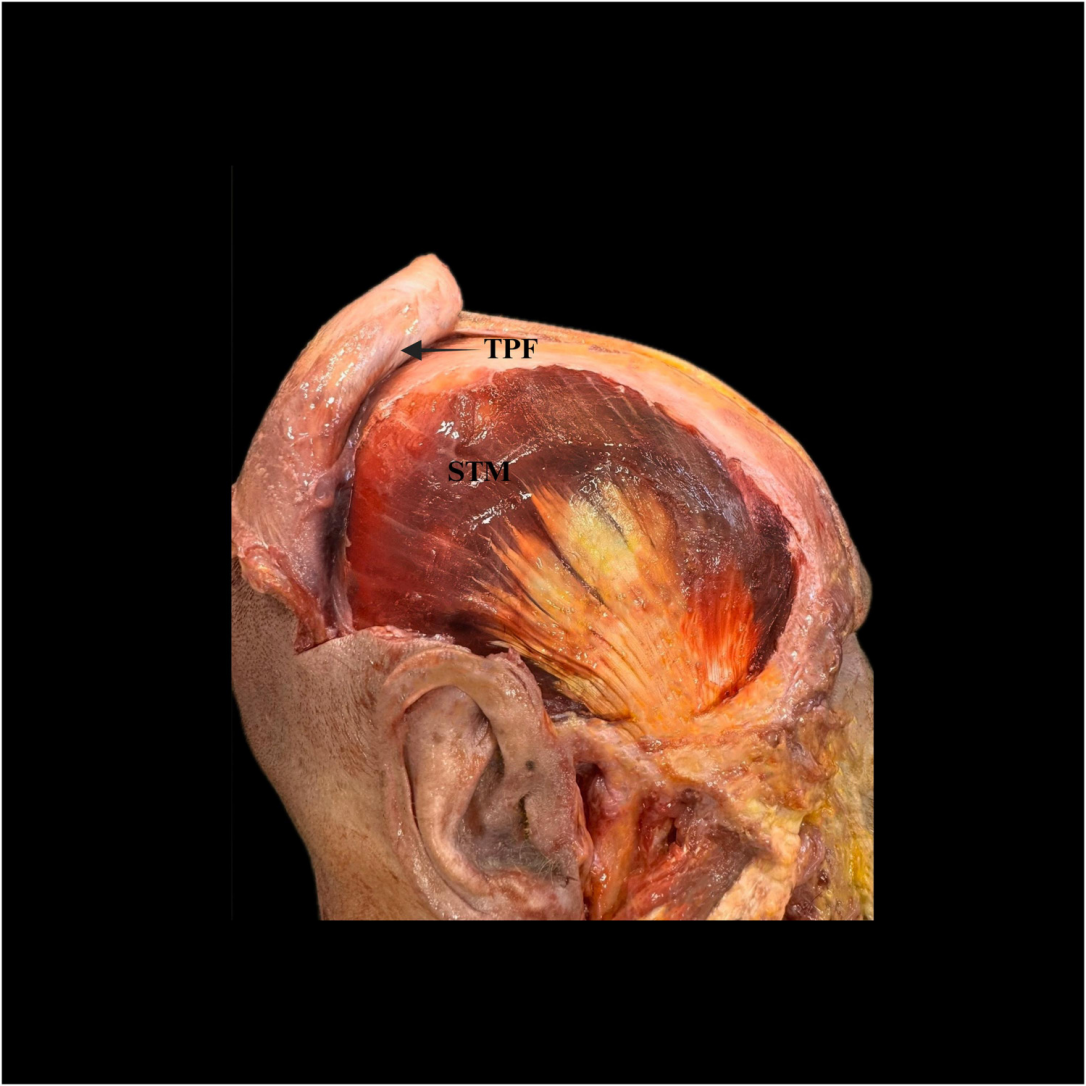
Open dissection of the temporal region relevant to temporalis muscle flap (TMF) harvest. The temporoparietal fascia (TPF) is reflected anteriorly (arrow), exposing the superficial portion of the temporalis muscle (STM). This view demonstrates the sub-TPF loose areolar plane used for safe elevation of the TMF. TPF, temporoparietal fascia; STM, superficial temporalis muscle.

The layered anatomy and the correct sub-TPF dissection plane are illustrated in Figure 3.

Among these layers, the DTF is a critical protective landmark during elevation, shielding underlying neurovascular structures. Misidentifying or violating this plane can precipitate hematoma, seroma, ischemia or neural injury (8, 10). Preserving the loose areolar sub-TPF plane maintains anatomic integrity and protects the frontal-branch zone and the superficial temporal vessels (see Figure 3).

### 4.2 Critical analysis of the vascular anatomy of the temporalis muscle flap

Intraoperative safety hinges on meticulous identification and preservation of the anterior and posterior deep temporal pedicles (see §3.1); in patients with altered vascular anatomy or post-radiotherapy fibrosis, preoperative CTA/MRA mapping and gentle intramuscular dissection along vascular territories help mitigate ischemic risk (4, 8)

### 4.3 Dissection and insetting

Dissection and insetting (brief). Through a temporal or hemicoronal incision, elevate the flap in the sub-TPF loose areolar plane to protect the frontal branch and the superficial temporal vessels (see Figure 3). Incise the deep temporal fascia at the superior border of the zygomatic arch and elevate the temporalis on its anterior and posterior deep temporal pedicles, using gentle intramuscular dissection parallel to muscle fibers to preserve perforators (see Section 3.1). Additional reach can be obtained by releasing posterior attachments and, when indicated, detaching from the coronoid with tendon lengthening; avoid wide subperiosteal dissection near the coronoid to reduce trismus risk (see Section 5.4). Create a low-resistance tunnel to the recipient site—preauricular/subcutaneous for maxillary or nasopharyngeal access, or a transorbital corridor for periorbital/skull-base defects—using endoscopy-assisted visualization in narrow passages (see Figure 1 and Section 7.3). When split TMF is planned for parallel reconstructive sites, respect pedicle-based territories and confirm perfusion mapping preoperatively (see Section 3.4). Inset the muscle with tension-free, watertight mucosal closure and secure it to stable periosteal margins to obliterate dead space; consider quilting sutures or a bolster as needed. Place a closed-suction drain at the donor site and close in layers. To mitigate temporal hollowing, prioritize muscle-sparing elevation and consider immediate fat grafting or a patient-specific implant in selected patients (see Section 5.3). Initiate early jaw physiotherapy and monitor maximal interincisal opening (MIO) postoperatively according to the outcome schedule (see Section 5.2).

### 4.4 Surgical considerations and anatomical risks associated with zygomatic arch osteotomy

The inferior reach of the TMF is limited by the rigid anatomical boundary of the zygomatic arch. In selected cases, partial osteotomy of the arch may be required to achieve sufficient flap mobility and tension-free inset (10, 20). However, this maneuver introduces potential risks, including injury to the vascular pedicles or adjacent neural structures.

To mitigate these risks, careful preoperative planning with CTA and intraoperative osteotomy control are essential. Bone fragments should be preserved and repositioned with stability to maintain both anatomical integrity and facial contour (7, 20).

### 4.5 Anatomical considerations at the mandibular attachment of the temporalis muscle

The insertion of the TM into the coronoid process of the mandible forms a robust tendinous anchor. Controlled subperiosteal detachment at this site is often necessary to gain flap mobility; however, excessive or uncontrolled detachment may compromise mandibular strength, especially in irradiated bone or osteoporotic patients (8, 10).

Proper muscular release using refined subperiosteal techniques is critical for balancing functional reach with structural preservation. Over-manipulation may predispose patients to fracture or impaired mandibular function (6, 20).

### 4.6 Implications of anatomical variability and recommendations for preoperative surgical planning

Substantial interindividual variation in both vascular and neural anatomy within the temporal region necessitates personalized preoperative planning. Imaging modalities such as CTA and MRI are invaluable in delineating vascular branching patterns, muscle volume, and fascial integrity (7, 21, 22). Additionally, intraoperative electromyographic monitoring provides real-time feedback during nerve-sparing dissections, reducing the risk of injury to the FBFN (15, 19).

Routine incorporation of these technologies into preoperative workflows improves anatomical accuracy, increases flap viability, and enhances patient safety especially in reoperations or complex oncologic reconstructions (6, 8).

Table 3 summarizing critical anatomical considerations.

**Table 3:** Critical Anatomical Considerations and Clinical Relevance in TMF Harvesting.

Anatomical Structure	Surgical Significance and Recommended Management	Potential Complications if Mismanaged
Frontal branch of facial nerve	Meticulous superficial plane dissection (TPF/DTF interface)	Brow ptosis, impaired forehead elevation, asymmetry
Deep temporal fascia (DTF)	Robust barrier and key landmark for deep structure protection	Hematoma, seroma, flap ischemia, nerve injury
Dual vascular pedicles	Careful preservation of anterior/posterior deep temporal arteries	Flap ischemia, necrosis, compromised viability
Zygomatic arch osteotomy	Controlled partial osteotomy, careful bone repositioning	Vascular compression, nerve stretch, ischemia
Mandibular coronoid attachment	Gentle subperiosteal detachment, maintain mandibular integrity	Mandibular fracture, instability, compromised healing

## 5 Outcomes and clinical results

### 5.1 Flap survival and reliability

The TMF remains a cornerstone in craniofacial reconstruction due to its dependable vascular anatomy, characterized by dual independent pedicles that ensure robust perfusion even in compromised surgical fields (4, 13). Clinical data consistently affirm near-total flap survival rates across a wide spectrum of indications.

In a pivotal study involving 182 patients, Clauser et al. (13) reported a 100% flap survival rate in various craniofacial reconstructions. Similar findings were echoed by Shanmugan et al. (25), who documented complete flap viability in head and neck reconstructions. A recent systematic review by Laloze (8) reaffirmed these findings, highlighting minimal risk of total flap failure even in previously irradiated tissues.

### 5.2 Outcomes and assessment framework (for TMF)

Primary endpoints (indication-specific).

- Palatomaxillary/intraoral defects: fistula-free closure at 6 months (no oronasal leakage on clinical exam or dye test; no dehiscence requiring reoperation) (8, 23).
- Periorbital/orbital lining: stable, epithelialized lining at 6 months without breakdown, infection, or surgical revision (23).
- Skull-base/dural coverage: absence of CSF leak at 3 months, confirmed clinically ± endoscopy/imaging when indicated.

Secondary endpoints.

- Function: speech intelligibility and swallowing/aspiration status assessed by speech-language pathology; instrumented studies as needed (VFSS/FEES) (26).
- Mouth opening (trismus): maximal interincisal opening (MIO, mm) measured with standardized calipers/ruler protocol (report with mean ± SD and proportion <35 mm) (8).
- Donor-site aesthetics: temporal contour/hollowing on standardized photographs ± 3D surface scan or MRI/US volumetry; optional blinded panel rating or FACE-Q modules (27).
- Complications: hematoma, infection, wound issues; optionally grade using Clavien–Dindo to improve comparability (28).
- Resource use: operative time, length of stay, ICU/HDU requirement, 30/90-day readmissions and reoperations.
- Patient-reported outcomes (PROs): validated instruments such as UW-QOL (29) and EORTC QLQ-H&N35 (30), selected according to indication.

Assessment methods and timepoints.

- Clinical exam & endoscopy: leak testing for intraoral defects; nasal endoscopy where relevant (23).
- Instrumented studies: VFSS/FEES for swallowing; standardized MIO for trismus (8, 26).
- Imaging (as needed): US/CT/MRI for suspected collections, flap congestion, or donor-site volume change.
- Photography/3D: reproducible views/lighting to document temporal contour change.
- PROs: administer at baseline, 3–6 months, and 12 months.
- Suggested schedule: POD 3–7, 6–12 weeks, 3–6 months, 12 months (extend for comparative studies).

Reporting guidance.

Pre-specify one primary endpoint per indication cohort and report with 95% CIs; treat others as secondary with predefined timepoints. When comparing with free flaps, adjust for irradiation status, defect class/size, age/comorbidity, and center experience to limit confounding (8). A concise, indication-specific checklist is provided in Supplementary Table S1.

### 5.3 Functional outcomes

TMF achieves high functional efficacy, particularly when tailored to the anatomical requirements of the defect. In reconstructions of the oral cavity and palate, it supports restoration of key functions such as speech articulation and swallowing. Brennan et al. (23) reported 100% fistula-free outcomes post-palatal reconstruction, while Hassanein (31) observed substantial functional improvement in speech and deglutition in palatomaxillary defects.

In facial reanimation, TMF tendon transfers offer a reliable alternative to free-muscle flaps such as the gracilis. Although microvascular techniques provide superior outcomes in terms of emotional expressivity, TMF transfers deliver satisfactory results with lower surgical complexity. Boahene (32) reported notable improvements in oral commissure mobility and symmetry, with high patient-reported satisfaction. However, emotional spontaneity remains a challenge, as emphasized by Oyer et al. (24).

### 5.4 Cosmetic outcomes and donor-site morbidity

Aesthetically, the TMF offers advantages due to its proximity to recipient sites, eliminating the need for distant donor scars. Nonetheless, donor-site morbidity particularly temporal hollowing remains a frequent issue, observed in approximately 50%–75% of cases (8, 23). This is primarily due to muscle atrophy following harvesting.

Mitigation strategies such as autologous fat grafting and patient-specific polyetheretherketone (PEEK) implants have been effective in restoring contour and improving patient satisfaction (9, 10). Minimally invasive or muscle-sparing harvesting techniques have also shown promise. Tauro et al. (33) documented improved cosmetic outcomes and reduced scar visibility using combined coronal–intraoral approaches.

### 5.5 Complications and management strategies

Despite the overall reliability of the TMF, certain complications persist. Temporal hollowing remains the most prevalent aesthetic concern and is effectively addressed with secondary corrective procedures. Trismus is reported in 30%–40% of cases but typically resolves with conservative physiotherapy within weeks to months (8, 25).

Transient neuropraxia of the FBFN occurs in approximately 5%–25% of patients, with permanent deficits being rare thanks to modern surgical refinements and intraoperative neuromonitoring (19). Minor complications such as seromas, hematomas, and superficial infections are uncommon and generally self-limiting (34). Long-term deficits in mastication or jaw mobility are rare due to contralateral muscular compensation, with persistent trismus occurring in <5% of cases on long-term follow-up (34).

### 5.6 Comprehensive summary of clinical outcomes

Table 4 summarizes key clinical studies evaluating the TMF in terms of flap reliability, functional and cosmetic performance, and complication rates.

**Table 4:** Clinical outcomes of TMF reconstruction with corresponding levels of evidence.

Author (year)	No. of patients	Defect type	Flap success (%)	Major complications observed	Level of evidence (LoE)
Clauser et al. (1995) (13)	182	Craniofacial defects	100%	Temporal hollowing, transient trismus	III (Retrospective comparative study)
Brennan et al. (2017) (23)	Systematic Review	Palatal defects	100%	Temporal hollowing, transient trismus	II (Systematic review)
Hassanein (2017) (22)	32	Palatomaxillary defects	96.9%	Transient trismus, minor seromas	III (Retrospective study/case series)
Laloze et al. (2019) (8)	Systematic Review	Various craniofacial defects	Near 100%	Temporal hollowing (50%–75%), transient trismus	II (Systematic review)
Oyer et al. (2018) (24)	Retrospective	Facial reanimation	Comparable to free flaps	Transient trismus, minor seromas	III (Retrospective comparative study)

### 5.7 Critical analysis and recommendations

Clinically, the TMF continues to exhibit unparalleled reliability, consistently favorable functional outcomes, and a manageable complication profile, particularly advantageous when compared with more technically demanding microvascular free flap reconstructions. Shorter operative durations, reduced technical complexity, and predictable recovery trajectories represent key clinical advantages, significantly enhancing postoperative recovery and patient satisfaction (8, 25). Nonetheless, aesthetic and functional limitations such as donor-site temporal hollowing, reduced emotional spontaneity in dynamic reconstructions, and transient postoperative trismus highlight the need for continuous surgical refinement. Contemporary surgeons should prioritize minimally invasive harvesting techniques, proactive donor-site management through adjunctive cosmetic strategies (e.g., immediate fat grafting, customized PEEK implants), and individualized preoperative planning employing advanced imaging modalities such as CTA and MRI (8).

Future directions should emphasize prospective comparative clinical trials and further innovations in minimally invasive techniques and bioengineered adjuncts, with the aim of further improving outcomes and expanding TMF indications.

## 6 Comparison with alternative techniques

### 6.1 TMF vs. free tissue transfer (FTT)

FTT, including radial forearm flap, anterolateral thigh flap, fibula free flap, and rectus abdominis flap, is regarded as the gold standard for extensive craniofacial reconstructions, particularly when precise anatomical restoration is required. These techniques excel in providing anatomically matched, like-for-like tissue replacements (35, 36). Clinically, free flaps achieve superior outcomes, especially in extensive composite defects resulting from oncologic resections or trauma (13, 35).

Despite these advantages, FTT entails significant complexity, long operative times, and the need for microvascular anastomosis, which carries the risk of thrombosis (3%–10%) and may require urgent reoperation (36, 37). In contrast, TMF offers reduced operative duration, technical simplicity, and minimal flap failure risk. Its dual blood supply from the anterior and posterior deep temporal arteries ensures reliable perfusion, even in irradiated or previously operated tissuesb (4, 5). TMF is effective in reconstructing moderate-sized defects, including the orbit, midface, skull base, and intraoral regions. Clauser et al. (13) reported 100% survival in a series of 182 TMF procedures, while Brennan et al. (23) confirmed high success rates in palatal reconstructions.

TMF is especially suitable for patients with elevated surgical risk (e.g., elderly, vessel-depleted neck, radiation injury) (8, 25). Although it cannot match FTT in volume and complexity, TMF remains a dependable option for select moderate defects.

### 6.2 TMF vs. other regional flaps

Other regional flaps, such as the pectoralis major myocutaneous flap (PMMF), trapezius flap (TF), and latissimus dorsi flap (LDF), are used when FTT is contraindicated. PMMF provides reliable tissue bulk but often results in excessive volume, visible scarring, and compromised aesthetics in midface reconstructions (25, 36, 37).

TMF provides significant aesthetic advantages due to its proximity to the defect and reduced donor-site morbidity. It yields enhanced contouring and symmetry, particularly in midface and orbital reconstructions (8, 35). Comparative studies show higher cosmetic satisfaction with TMF compared to PMMF or TF (37).

Unlike supraclavicular or infrahyoid flaps, TMF provides more reliable vascularization and ease of harvesting. However, its limitations include insufficient reach for defects below the mandible or in the cervical/esophageal region, where PMMF or LDF are preferred (35, 37). In palatal reconstruction, TMF outperforms obturators, which lack tissue integration and require maintenance. TMF provides permanent closure and improved functional and quality-of-life outcomes (35, 37).

### 6.3 Comprehensive comparative summary and recommendations

Table 5 summarizes comparative clinical indications, advantages, disadvantages, and operative complexity of TMF vs. alternative reconstructive methods. TMF remains a vital option, especially in anatomically or clinically challenging scenarios.

**Table 5:** Comparative outcomes of temporalis muscle flap and alternative reconstruction techniques.

Technique	Clinical indication	Advantages	Disadvantages	Operative complexity
TMF	Orbit, midface, oral cavity, skull base	Shorter operative time, low flap failure risk, excellent midface aesthetics	Temporal hollowing, limited bulk, not suitable below mandible	Moderate (non-microsurgical)
FTT	Extensive head and neck defects	Tissue versatility, anatomical precision	Long operative time, vascular complications	High (microsurgical)
PMMF	Lower face, extensive neck defects	High tissue bulk, robust vascular supply	Prominent scarring, high donor-site morbidity	Moderate (non-microsurgical)
Supraclavicular & infrahyoid flaps	Small-to-medium defects	Reduced morbidity, good cosmetic outcomes	Limited reach, insufficient for large defects	Moderate (non-microsurgical)
Dental obturators	Palatal defects	Immediate coverage, non-surgical	Hygiene management, no vascularized tissue	Low (prosthetic only)

Clinically, TMF remains critically important, particularly in patients unsuited for prolonged microsurgery or complex free flap reconstruction. Surgeons must carefully balance defect size, patient comorbidities, anatomical considerations, and long-term outcomes to select the most appropriate reconstructive option.

Continued refinement of minimally invasive harvest techniques, adjunctive cosmetic procedures to mitigate donor-site morbidity, and comparative outcomes research remain essential to further optimize TMF's clinical efficacy and patient satisfaction.

For high-yield decision points, see Clinical Box 1.

Clinical Box 1 **When the Temporalis Muscle Flap (TMF) “wins”**

**Table:** 

Key scenarios and quick tips
A. Irradiated palatomaxillary defect in an older, comorbid patient Why TMF: vascularized, reliable closure with shorter OR time vs. many free flaps — practical in frail or vessel-depleted patients (23, 37). Clinical note: obturators can restore function but may underperform for speech/leakage in larger defects; flap reconstruction can improve QoL in selected cases. Caveat: when substantial bony support is required, combine TMF with prosthetic/osseous solutions or consider a bony free flap.
B. Endoscopic periorbital reconstruction after orbital exenteration Why TMF: endoscopy-assisted TMF enables low-morbidity inset with minimal scarring and uncomplicated healing in selected cases (38). Tip: plan a concealed temporal entry and maintain the sub-TPF plane to protect the frontal branch.
C. Skull-base/dural reconstruction *via* transorbital route Why TMF (deep temporal myofascial variant): provides robust, vascularized coverage when free tissue is impractical; early reports support reach to anterior/middle cranial fossa with minimal access (6, 7). Tip: obtain preoperative imaging to map deep temporal pedicles; consider limited sphenoid drilling to extend arc of rotation.

### 6.4 Feasibility, learning curve, and complications — temporalis muscle flap (TMF) vs. free flaps

Operative feasibility. TMF reconstruction avoids microvascular anastomosis, which typically shortens operative timeand reduces postoperative monitoring needs—a decisive advantage in frail, elderly, or vessel-depleted patients and when rapid, reliable soft-tissue obliteration is the primary goal (10, 39). In contrast, free flaps enable composite reconstruction (bone/skin/soft tissue) but require microsurgical expertise, longer operating times, and higher resource utilization (microscope, specialized team, ICU/HDU protocols) (39, 40). Accordingly, TMF is often preferred for moderate, non-osseous defects or in high-risk hosts, while free flaps remain the standard for large composite defects requiring bony support or external skin (40).

Learning curve. Open TMF elevation has a moderate learning curve grounded in precise knowledge of the sub-TPF plane and pedicle preservation; endoscopic/ETO TMF adds technical complexity and equipment demands and should be adopted with structured training and proctorship (38, 41, 42). Microsurgical free-tissue transfer retains a high, center-dependent learning curve, with outcomes linked to institutional volume, team coordination, and streamlined perioperative pathways (40).

Complication profile. TMF rarely fails from vascular causes; principal risks are trismus (after over-aggressive subperiosteal elevation near the coronoid) and temporal hollowing/contour change the latter mitigable with muscle-sparing elevation, immediate fat grafting, or PEEK implants (8, 13, 21, 22, 43, 44). Free flaps carry risks of anastomotic thrombosis with partial/total flap loss and donor-site morbidity specific to the tissue harvested; salvage is time-critical and resource-intensive (39, 40).

Decision framework. Selection should be indication-driven and explicitly balance resources, patient risk, and functional/aesthetic goals. High-yield scenarios favoring TMF are summarized in Clinical Box 1; a structured head-to-head overview is provided in Table 6. For endoscopic indications see §7.3, and for planned muscle splitting across separate reconstructive sites see §3.4 (10, 17, 18).

**Table 6:** TMF vs free flaps — feasibility, learning curve, and complications.

Dimension	Temporalis muscle flap (TMF)	Free flaps	Implication/when to prefer
Operative time	Shorter; no microvascular anastomosis.	Longer; requires microvascular anastomosis.	TMF in frail/high-risk or time-sensitive settings.
Resources & logistics	Standard instruments; optional endoscopic set; no micro team.	Microsurgical team, microscope, ICU/HDU monitoring often needed.	TMF suits resource-constrained environments.
Learning curve	Moderate (open); higher for endoscopic/ETO harvest.	High; center- and operator-dependent.	Match complexity to team experience.
Monitoring & salvage	Clinical monitoring; low salvage complexity; rare vascular failure.	Intensive monitoring; time-critical salvage for thrombosis.	Lower monitoring burden favors TMF when feasible.
Donor-site morbidity	Temporal hollowing/contour change; mitigable (muscle-sparing, fat grafting, PEEK).	Flap-specific donor issues (forearm/thigh/leg).	Choose per patient priorities and mitigation options.
Complications	Trismus if over-elevated near coronoid; sensory changes possible.	Partial/total loss from anastomotic thrombosis; flap-specific risks.	Balance vascular risk vs functional/aesthetic goals.
Hospital stay	Often shorter; limited monitoring needs.	Often longer; ICU/HDU pathways.	Impacts cost and bed turnover.
Functional goals	Reliable lining/obliteration for moderate soft-tissue defects.	Composite reconstruction (bone/skin/soft tissue).	TMF for moderate non-osseous; free flaps for large composite.
Aesthetic footprint	Hidden temporal incision; risk of hollowing (preventable).	Donor scars vary by flap (forearm/thigh/leg).	Set expectations; plan prevention strategies.
Typical indications	Irradiated or vessel-depleted fields; elderly/comorbid; need for rapid reliable coverage.	Large composite defects needing bone/skin island; external resurfacing.	Use type-by-indication algorithm (see Clinical Box 1).

## 7 Future directions

### 7.1 Donor-site morbidity

Temporal hollowing remains the key aesthetic concern, driven by anterior volume loss and inadequate fascial support. MRI-based planning enables individualized prevention. Partial muscle-sparing harvests, immediate fat grafting, and patient-specific PEEK implants show promise, but require prospective validation for durability and cost-effectiveness (13, 21, 22, 43, 44).

### 7.2 Functional restoration

Classic temporalis transfers provide reliable motion but limited spontaneity. Cross-facial nerve grafts and facial–temporal nerve coaptation may restore emotion-driven activation; studies should standardize timing, feasibility mapping, and long-term neuromuscular outcomes (32, 45). Future research priorities are summarized in (Table 7).

**Table 7:** Future directions and research priorities for the temporalis muscle flap (TMF).

Theme	What we know	What to study next
Donor-site morbidity	Temporal hollowing driven by anterior volume loss; prevention with muscle-sparing harvests, immediate fat grafting, and PEEK implants.	Prospective trials on durability, aesthetic stability, and cost-effectiveness.
Functional restoration	Transfers are reliable but show limited spontaneity; cross-facial grafts and facial–temporal coaptation are promising.	Standardized protocols and long-term neuromuscular outcomes.
Minimally invasive/ETO	Endoscopic harvest reduces soft-tissue trauma and helps protect FBFN/STA; early series are favorable.	Multicenter comparisons vs open harvest; learning curve and indications.
Role vs free flaps	TMF advantageous for moderate defects, irradiated/vessel-depleted fields, and high-risk patients.	Registries/pragmatic trials on outcomes, QoL, and cost.

See Clinical Box 1 for quick, type-by-indication scenarios; §7.3 for endoscopic variants; and §3.4 for split-TMF prerequisites.

### 7.3 Minimally invasive techniques

Endoscopic/ETO harvest can reduce soft-tissue trauma while protecting the frontal branch and superficial temporal vessels. Early results are favorable; multicenter comparative studies are needed to define indications, learning curve, and safety vs. open harvest (38, 41, 42).

### 7.4 Role in the microsurgical era

Despite advances in free tissue transfer, TMF remains advantageous for moderate defects, irradiated or vessel-depleted fields, and high-risk patients. Registries and pragmatic trials should clarify where TMF outperforms free flaps on time-to-treatment, complications, cost, and quality of life (39, 40, 46).

## 8 Conclusion

The temporalis muscle flap (TMF) remains a highly valuable and versatile option in modern craniofacial reconstruction, owing to its robust dual vascular anatomy, anatomical proximity to midfacial and skull base defects, and reliability in compromised surgical fields. Despite limitations such as temporal hollowing and limited reach below the mandible, these challenges can be effectively addressed through refined surgical techniques and adjunctive aesthetic strategies.

This review consolidates anatomical and surgical evidence demonstrating TMF's unique utility in cases where microvascular free tissue transfer is contraindicated, including patients with comorbidities, irradiated tissues, or vessel-depleted anatomy. The integration of advanced MRI protocols into clinical practice is emphasized as a key strategy for improving flap planning, viability assessment, and outcome prediction.

Future research should focus on optimizing minimally invasive harvesting techniques, enhancing spontaneous function through nerve coaptation, and evaluating the clinical utility of bioengineered solutions. A systematic effort to standardize nomenclature and outcome metrics is also necessary to improve comparability and evidence synthesis.

By highlighting both enduring strengths and future opportunities, the TMF is reaffirmed as a critical and evolving tool in the reconstructive surgeon's practice, capable of adapting to contemporary challenges and diverse patient needs.
